# Melanization and Pathogenicity in the Insect, *Tenebrio molitor,* and the Crustacean, *Pacifastacus leniusculus,* by *Aeromonas hydrophila* AH-3

**DOI:** 10.1371/journal.pone.0015728

**Published:** 2010-12-29

**Authors:** Chadanat Noonin, Pikul Jiravanichpaisal, Irene Söderhäll, Susana Merino, Juan M. Tomás, Kenneth Söderhäll

**Affiliations:** 1 Department of Comparative Physiology, Uppsala University, Uppsala, Sweden; 2 National Center for Genetic Engineering and Biotechnology, National Science and Technology Development Agency, Bangkok, Thailand; 3 Departamento de Microbiología, Facultad de Biología, Universidad de Barcelona, Barcelona, Spain; Charité-University Medicine Berlin, Germany

## Abstract

*Aeromonas hydrophila* is the most common *Aeromonas* species causing infections in human and other animals such as amphibians, reptiles, fish and crustaceans. Pathogenesis of *Aeromonas* species have been reported to be associated with virulence factors such as lipopolysaccharides (LPS), bacterial toxins, bacterial secretion systems, flagella, and other surface molecules. Several mutant strains of *A. hydrophila* AH-3 were initially used to study their virulence in two animal species, *Pacifastacus leniusculus* (crayfish) and *Tenebrio molitor* larvae (mealworm). The AH-3 strains used in this study have mutations in genes involving the synthesis of flagella, LPS structures, secretion systems, and some other factors, which have been reported to be involved in *A. hydrophila* pathogenicity. Our study shows that the LPS (O-antigen and external core) is the most determinant *A. hydrophila* AH-3 virulence factor in both animals. Furthermore, we studied the immune responses of these hosts to infection of virulent or non-virulent strains of *A. hydrophila* AH-3. The AH-3 wild type (WT) containing the complete LPS core is highly virulent and this bacterium strongly stimulated the prophenoloxidase activating system resulting in melanization in both crayfish and mealworm. In contrast, the Δ*waa*E mutant which has LPS without O-antigen and external core was non-virulent and lost ability to stimulate this system and melanization in these two animals. The high phenoloxidase activity found in WT infected crayfish appears to result from a low expression of pacifastin, a prophenoloxidase activating enzyme inhibitor, and this gene expression was not changed in the Δ*waa*E mutant infected animal and consequently phenoloxidase activity was not altered as compared to non-infected animals. Therefore we show that the virulence factors of *A. hydrophila* are the same regardless whether an insect or a crustacean is infected and the O-antigen and external core is essential for activation of the proPO system and as virulence factors for this bacterium.

## Introduction

*Aeromonas hydrophila* is a Gram-negative bacterium living in aquatic environments. It can be found in freshwater, seawater, and also chlorinated-drinking water. This bacterium has been considered as a food-borne pathogen since it is found in many food products, for example sea food, shrimp cocktail, ground meat and raw vegetables [1]–[3]. *A. hydrophila* is the most common species of *Aeromonas* that causes infections in human and other animals such as amphibian reptile, fish and crayfish [4]–[6]. Infection of this bacterium is also a major problem in carp aquaculture in India [7]. Furthermore, *A. hydrophila* was isolated from several rainbow trout farms and found resistant to antibiotics used in aquaculture in Australia [8], and recently it was also isolated from freshwater crayfish (*Pacifastacus leniusculus*) and was found to be highly virulent to this animal [5].

The pathological conditions found in fish infected with *A. hydrophila* are usually hemorrhagic septicemias (reddish eyes, skin, gills, and fins) and tail and fin rot [9]. Catfish infected with this bacterium exhibited hemorrhagic fins and had larger spleen, kidney and liver [10]. Crayfish infected with *A. hydrophila* showed necrotic injury in gill, heart and hepatopancreas [5]. The pathogenesis of *Aeromonas* species have been reported to be associated with virulence factors such as lipopolysaccharides (LPS), bacterial toxins, bacterial secretory system, flagella and capsules. These factors are believed to be important in both resistance of bacteria to host immune responses and bacterial virulence [3], [6], [11]–[15]. Some other factors, such as siderophores (high-affinity iron chelating molecules) and porins (pore-forming proteins), are also reported to be involved in bacterial growth and *A. hydrophila* pathogenesis [16]–[18].

**Figure 1:**
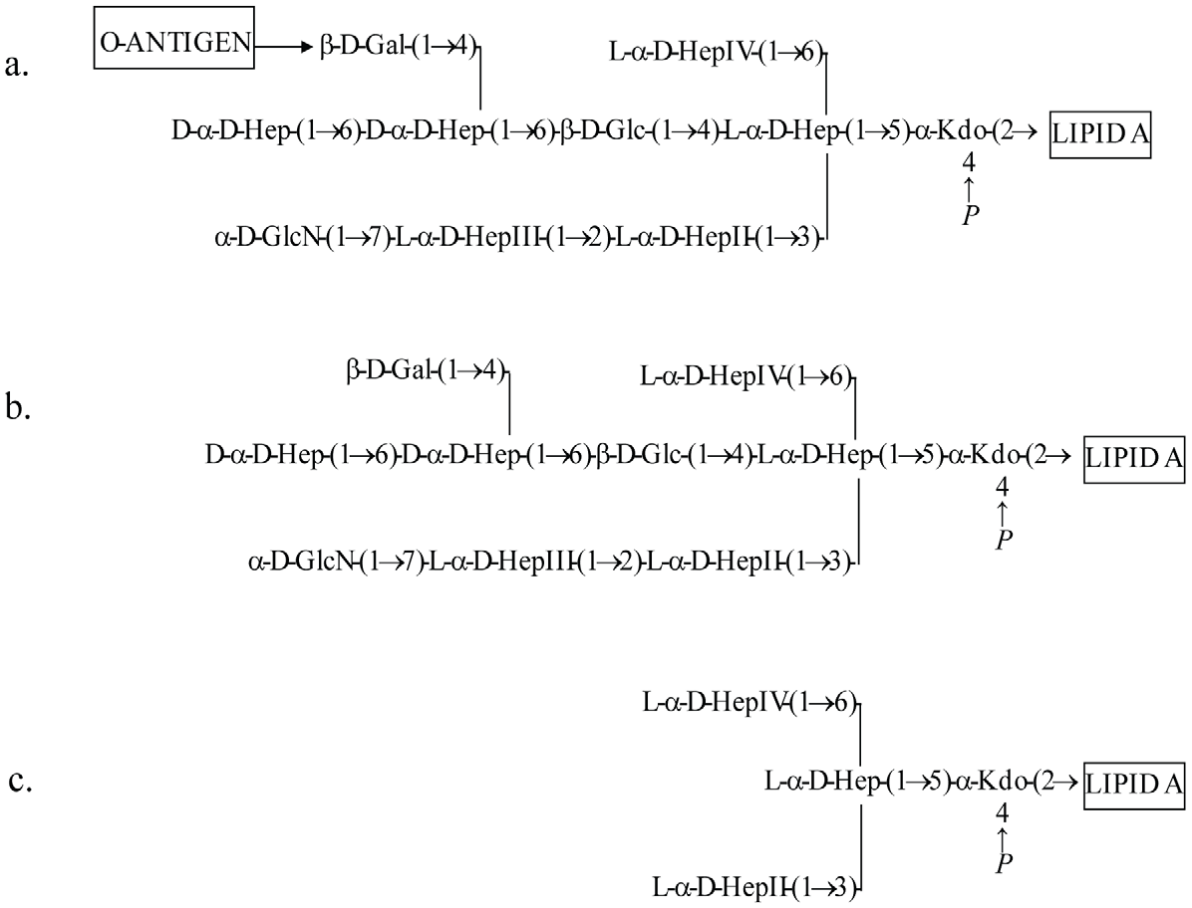
LPS structures of *A. hydrophila* AH-3 wild type (a), AH-3 Δ*waa*L mutant (b), and AH-3 Δ*waa*E mutant (c).

LPS have been widely studied and reported to contribute an important role in resistance of *Aeromonas* spp. to the host immune system as well as in inducing harmful effects to the host [19]–[21]. The O-antigen, LPS core (external and internal), and lipid A, are assembled to form a complete LPS structure and are considered to play a role in bacterial pathogenesis [20], [22]. Several genes of *A. hydrophila* involved in LPS biogenesis have been studied. For example, the *waa*L gene encodes a ligase protein required for ligation of the O-antigen to the LPS core, *wzz* gene is responsible for the length of the O-antigen, *waa*E gene plays a role in LPS core synthesis, and *msb*B gene is involved in lipid A biosynthesis. These genes are believed to be associated with virulence of *A. hydrophila* [22]–[24]. The chemical structure of the most relevant LPS structure from *A. hydrophila* AH-3 strain and mutants is shown in Figure 1.

Previously, crayfish has been used as an experimental model to study the host immune response after *A. hydrophila* challenge and melanization, e.g. phenoloxidase activity was found to be important for crayfish immune defense against *A. hydrophila* infection [5], [25]. The details of prophenoloxidase (proPO) activating cascade have been studied in *Manduca sexta* and *Tenebrio molitor*, and the induction of melanization in *Tenebrio* larvae can be easily observed after microbial infection [26], [27]. In this study we used *A. hydrophila* AH-3 wild type strain and several mutants on crayfish and *Tenebrio* experimental models to study the *A. hydrophila* most determinant virulence factor of in these animal models. Furthermore, we also studied the immune responses of the host to infection with *A. hydrophila* AH-3 virulent or non-virulent strains in both animal models.

## Results

### 1. Virulence of *A. hydrophila* AH-3 strains

To investigate how surface molecules on bacteria or secretion systems influence pathogenicity, the virulence of *A hydrophila* AH-3 wild type and several mutant strains was studied in two animal species, *P. leniusculus* (Table 1) and *T. molitor* (Table 2). These bacterial strains have been mutated in genes involved in the synthesis of cell surface structures and molecules, or secretion systems. No major differences in growth curves could be observed among the mutants when they grow in LB or in minimal Davies medium with glucose as a single carbon and energy source. In this study, *A. hydrophila* B1 was used as a positive control, since this strain was previously reported as a highly virulent bacterium to freshwater crayfish [5]. Since the bacterial species used in this study had mutations in genes encoding some component of the cell walls, this in some cases had effects on the character of the bacterial mutants, which made it difficult to adjust the bacterial dose for injection to be exactly the same for each mutant species. As a consequence, there are some variations in injected dose or CFU between different bacterial mutant strains as shown in Table 1 and Table 2. The virulence of all bacterial strains can be determined from the injected CFU and the time of death after bacteria injection. Similar results for the different strains were obtained in crayfish and mealworm. *A. hydrophila* B1 used as a positive control was the most virulent strain causing death within 4 h and 21 h in crayfish and mealworm, respectively. The *A. hydrophila* AH-3 WT was less virulent than *A. hydrophila* B1, and this wild-type strain caused death within 25 h in both crayfish and mealworm. *A. hydrophila* AH-3 which lacks a Type VI secretion system (T6SS) and the AH-3 strain which lacks lateral flagella [28] showed similar virulence as the *A. hydrophila* AH-3 WT. In contrast, the mutant strains AH-3 Δ*uge* and Δ*wzz* [24] seemed to be more virulent than the WT in crayfish since all challenged animals died especially if a lower CFU of these two mutants was used for challenge. However, infection with AH-3 Δ*uge* or Δ*wzz* mutants took approximately 60 h or 45 h to cause crayfish or mealworm death, respectively. This is about two-fold longer time when compared to the WT. Therefore, *A. hydrophila* AH-3 which lacks a Type III secretion system (T3SS) [29], the strain which lacks polar flagellum [30] and all other mutant strains, excluding the Δ*waa*E (O:34 antigen and external core negative strain) [23], were all virulent but to a lesser extent than the *A. hydrophila* AH-3 WT. The Δ*waa*E was not virulent at all and could not kill any animals within the experimental period in this study. Moreover, it is important to notice that AH-3 Δ*waa*L [23] mutant showed a decrease in virulence but not to the same extent as that of the AH-3 Δ*waa*E mutant. Therefore, the virulent *A. hydrophila* AH-3 WT and the non-virulent Δ*waa*E were selected for further detailed studies. The AH-3 Δ*waa*E mutant showed a generation time in rich or minimal medium never superior to 20% of the corresponding wild type strain AH-3.

**Table 1:** Virulence of different *A. hydrophila* AH-3 strains in *P. leniusculus* (crayfish).

Bacterial strain	Injection CFU/crayfish (×10^6^)	Mortality (No. dead/No. tested)	Time of dead after injection(h)
*A. hydrophila* B1	7.2±0.5	4/4	3.8±0.3
*A. hydrophila* AH-3 wild type	4.2±0.7	6/9	25.5±8.4
AH-3 T3SS negative	3.0±0.2	3/4	46.0±11.8
AH-3 T6SS negative	8.8±0.3	4/4	13.3±3.6
AH-3 Δ*waa*L O:34 LPS-antigen negative	2.8±0.2	2/7	90.0±2.0
AH-3 polar flagellum negative	2.2±0.3	5/9	57.8±17.9
AH-3 lateral flagella negative	6.2±0.6	7/9	25.3±6.4
AH-3 Δ*waa*E O:34 antigen and LPS core negative	7.9±0.5	0/7	-
AH-3 Δ*msb*B lipid alteration	3.0 ±0.7	3/4	37.3±17.0
AH-3 major porin negative	3.0±0.4	3/7	97.7±24.3
AH-3 siderophore negative	1.4±0.1	3/4	42.3 16.5
AH-3 *uge* lacks capsule	2.3±0.0	4/4	59.6±16.3
AH-3 *wzz* lacks some repetitions of the O:34 antigen LPS	1.4±0.3	4/4	59.8±11.0

Mutant strains used:

AH-3 lateral flagella negative: AH-3:lafK [28], AH-3 T3SS negative: A3::*axsA* [29], AH-3 T6SS negative: AH3 Δ*vasH* (this work), AH-3Δ*waa*L [23], AH-3 polar flagellum negative: A3:flrA [30], AH-3Δ*waa*E [23], AH-3Δ*msbB* (this work), AH-3 major porin negative: AH-330 [17], AH-3 siderophore negative: AH-3:: *CirA* (this work), AH-3 lacking capsule: A3:: *uge* (this work), A3::*wzz* [24].

**Table 2:** Virulence of different *A. hydrophila* AH-3 strains in *T. molitor* larvae (mealworm).

Bacterial strain	Injection CFU/mealworm (×10^4^)	Mortality (No. dead/No. tested)	Time of dead after injection(h)
Ringer's solution	-	7/40	7 days
*A. hydrophila* B1	1.3	10/10	16–21
*A. hydrophila* AH-3 wild type	1	20/20	21–25
AH-3 T3SS negative	1.1	10/10	41
AH-3 T6SS negative	1.8	10/10	21
AH-3 Δ*waa*L O:34 LPS-antigen negative	0.8	10/10	4–7 days
AH-3 polar flagellum negative	0.4	10/10	20–21
AH-3 lateral flagella negative	0.7	10/10	20
AH-3 Δ*waa*E O:34 antigen and LPS core negative	1.7	2/20	2–3 days
AH-3 Δ*msb*B lipid alteration	0.4	10/10	42
AH-3 major porin negative	0.6	10/10	46
AH-3 siderophore negative	0.7	10/10	46
AH-3 *uge* lacks capsule	1	10/10	46
AH-3 *wzz* lacks some repetitions of the O:34 antigen LPS	0.7	10/10	45

Mutant strains used:

AH-3 lateral flagella negative: AH-3:lafK [28], AH-3 T3SS negative: A3::*axsA* [29], AH-3 T6SS negative: AH3 Δ*vasH* (this work), AH-3Δ*waa*L [23], AH-3 polar flagellum negative: A3:flrA [30], AH-3Δ*waa*E [23], AH-3Δ*msbB* (this work), AH-3 major porin negative: AH-330 [17], AH-3 siderophore negative: AH-3:: *CirA* (this work), AH-3 lacking capsule: A3:: *uge* (this work), A3::*wzz* [24].

### 2. Cytotoxicity of extracellular products of bacteria

**Figure 2:**
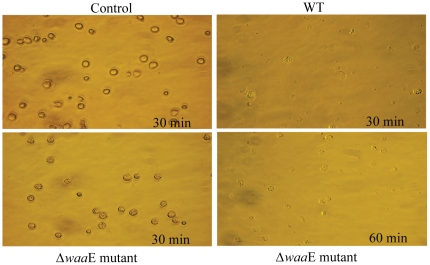
Cytotoxicity of extracellular products of *A. hydrophila* AH-3 wild type and Δ*waa*E mutant. Crayfish hpt cells were incubated with sterile-filtered culture medium from overnight grown bacteria or with fresh medium used as a control. Cell morphology was observed every 15 min for 2 h. This experiment was repeated 2 times.

To investigate whether the toxins or extracellular products produced from the highly virulent *A. hydrophila* WT and the non-virulent Δ*waa*E play a role in pathogenicity the bacterial extracellular products were prepared from these two strains and were used for cytotoxicity test using the crayfish hematopoietic cells (Figure 2). Extracellular products from both bacterial strains exhibited cytotoxic effects to the cells. Hematopoietic cells were completely lysed within 30 and 60 min after incubation with the extracellular products from the *A. hydrophila* WT and the Δ*waa*E, respectively.

### 3. Bacterial clearance in mealworms

**Figure 3:**
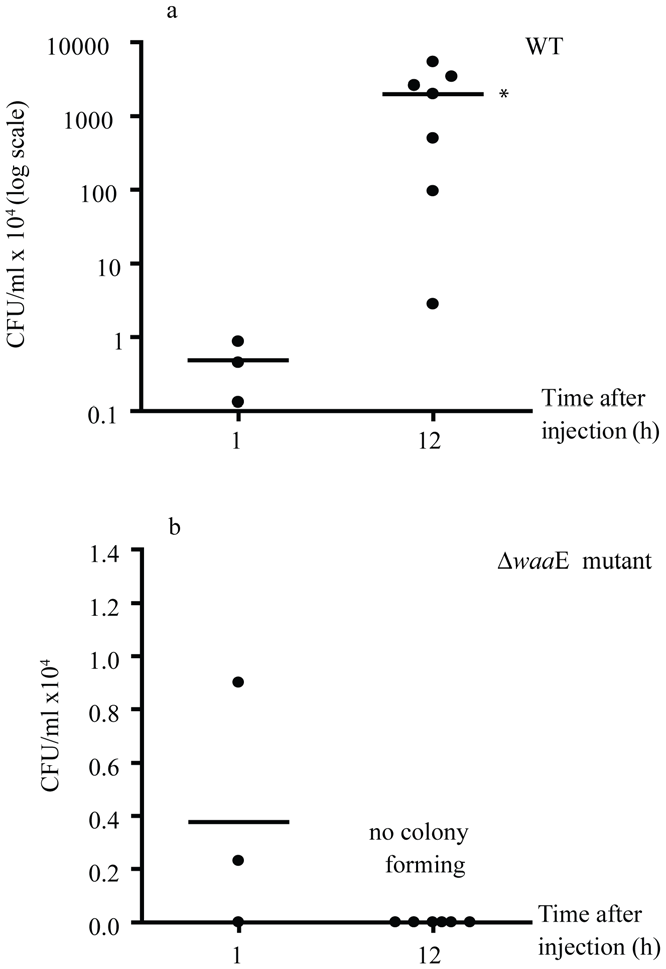
Bacterial clearance in mealworm. *A. hydrophila* AH-3 WT (a) or Δ*waa*E mutant (b) were injected into the worm at amounts of 0.4–0.6×10^4^ CFU or 1.8–2.9×10^4^ CFU, respectively. Hemolymph samples were collected at 1 h (n = 3 for each group) and 12 h (n = 7 for each group) after injection for determining CFU of bacteria. This experiment was repeated 2 times. • represents CFU of each individual. – represents mean CFU at each time point. * *P*<0.05, significant difference when compared to mean CFU at 1 h. Statistical analysis was performed using T-test.

The results in Figure 3 clearly show that mealworm could not eliminate the virulent AH-3 WT from their hemolymph and the bacteria grew very fast at 12 h after injection, from 0.4–0.6×10^4^ CFU/animal at the time of injection to an average of 2×10^7^ CFU/ml hemolymph (Figure 3a). In contrast, the worms completely cleared the non-virulent Δ*waa*E mutant within 12 h after injection, and no bacterial colonies were observed after 12 h injection of 1.8–2.9×10^4^ CFU/animal (Figure 3b).

### 4. Effect of bacterial challenge on the expression of crayfish antimicrobial peptides

**Figure 4:**
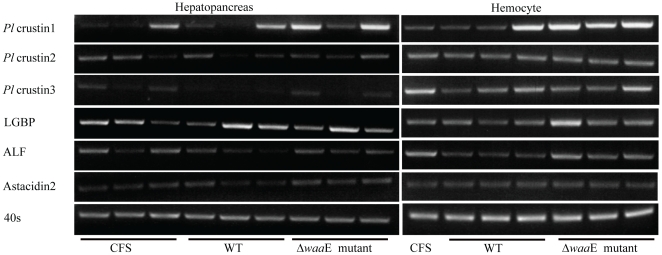
Expression of AMP genes in crayfish hepatopancreas and hemolymph after bacterial challenge. Hepatopancreas and hemolymph were taken out from the crayfish at 6 h after bacterial injection. Five hundred and 100 ng of total RNA from hepatopancreas and hemocytes, respectively, were used for RT-PCR. The PCR product of each gene was detected at the following cycles: 25 cycles for 40S; 30 and 25 cycles for *Pl*crustin1 in hepatopancreas and hemocyte, respectively; 28 and 22 cycles for *Pl*crustin2 in hepatopancreas and hemocyte, respectively; 30 cycles for *Pl*crustin3, LGBP, ALF and astacidin2. Each lane shows gene expression in each crayfish.

The virulent and non-virulent *A. hydrophila* strains were tested to see whether they have any effect on the expression of antimicrobial peptide genes in crayfish. The expression levels of crayfish antimicrobial peptide genes were determined 6 h after injection of *A. hydrophila* WT and the Δ*waa*E mutant. The results in Figure 4 show that there was no change in expression level of *Pl*crustin1 in hepatopancreas or hemocytes after injection with WT, but the expression of this gene was increased in both tissues of crayfish injected with the Δ*waa*E mutant strain. *Pl*crustin2 expression was not changed after injection with WT or the Δ*waa*E mutant. In the case of *Pl*crustin3, expression of this gene in hepatopancreas was decreased after injection with WT, but the expression was not changed after injection with Δ*waa*E mutant. The expression level of LGBP did not seem to be changed after either injection of WT or Δ*waa*E mutant. The ALF gene expression was obviously decreased at least in hemocytes following AH-3 WT injection, but not changed after Δ*waa*E mutant injection while the expression of astacidin2 was not changed after injection with either AH-3 WT or Δ*waa*E mutant.

### 5. Induction of melanization and activation of the proPO system

**Figure 5:**
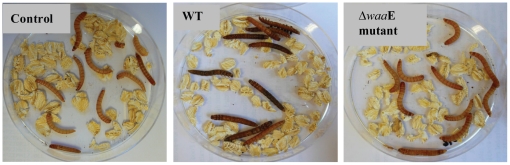
Melanization in mealworm. Mealworms were injected with *A. hydrophila* AH-3 WT (1×10^4^ CFU) or Δ*waa*E mutant (1.7×10^4^ CFU) (10 animals for each group), and melanin formation was observed for 7 days or until the animal died.

The results presented in Figure 5 clearly show that melanin was formed in mealworms injected with the virulent AH-3 WT. On the other hand, melanin formation could not be observed in mealworms injected with control buffer or the non-virulent Δ*waa*E mutant strain.

**Figure 6:**
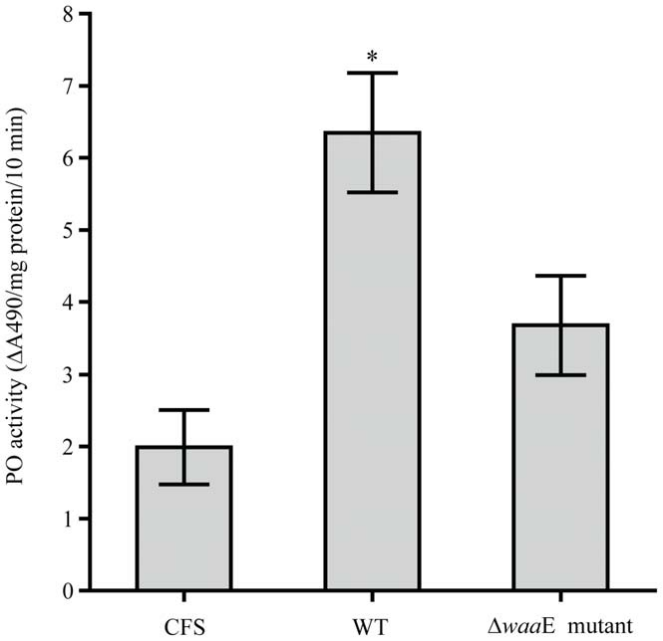
Activation of the proPO system in crayfish after bacterial challenge. Crayfish was injected with *A. hydrophila* AH-3 WT (5.8×10^6^ CFU) or Δ*waa*E mutant (10.1×10^6^ CFU) or CFS (control) (5 crayfish were used for each group). Six hours after injection, hemolymph was collected and used for HLS preparation and PO activity assay. The experiments were performed 2 times with a number of 10 crayfish for each experimental group. The results shown are mean of the 2 separate experiments. The bars represent SE of the data. * *P*<0.05, significant difference when compared to CFS. Statistical analysis was performed using one-way ANOVA followed by Turkey's multiple comparison test.

**Figure 7:**
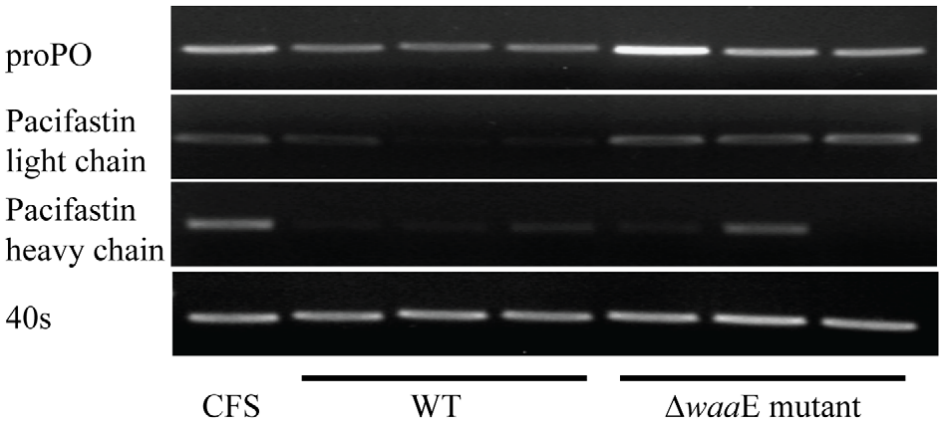
Expression of proPO and pacifastin transcripts. Hemolymph was taken out from the crayfish at 6 h after CFS or bacterial injection. One hundred of total RNA from hemocytes was used for RT-PCR. The PCR product of each gene was detected at the following cycles: 25 cycles for 40S and proPO, 30 cycles for pacifastin light chain, and 38 cycles for pacifastin heavy chain. Each lane shows gene expression in each crayfish.

Activation of the proPO system in crayfish after injection with the AH-3 WT or the Δ*waa*E mutant was studied and the results are shown in Figure 6. Activity of PO in a hemocyte lysate prepared from crayfish injected with AH-3 WT was significantly increased compared to the buffer injected control group (*P*<0.05). However, activity of PO was increased to a low extent in crayfish injected with the Δ*waa*E mutant. The proPO and pacifastin, a proPO activating enzyme inhibitor [31], transcripts were examined (Figure 7). The proPO and pacifastin light chain (proteinase subunit) transcripts were decreased in WT infected crayfish but there were no obvious changes in the Δ*waa*E mutant infected animals. However, both AH-3 WT and Δ*waa*E mutant showed a similar effect on the expression of the pacifastin heavy chain (transferrin subunit) [31] when compared to the CFS control animals.

## Discussion

*Aeromonas hydrophila* is a pathogenic bacterium for both terrestrial and aquatic animals. Several mutant strains of *A. hydrophila* AH-3 were used to study their virulence in two different animal species, *Pacifastacus leniusculus* (crayfish) and *Tenebrio molitor* larvae (mealworm). The *A. hydrophila* AH-3 strains used in this study have mutations in genes involved in the synthesis of flagella, LPS structures, secretion systems, and some other factors, which have been reported to be involved in *A. hydrophila* pathogenicity [6], [11]–[15].

Polar flagellum and lateral flagella are factors suspected to be involved in *Aeromonas* spp. virulence. Many studies show that mutation in genes involved in polar flagellum and lateral flagella syntheses lead to reduction in bacterial motility and adhesion of *Aeromonas* spp. to Hep-2 cell [11], [28], [30]. There is at least 50% of *Aeromonas* spp. which have two flagella systems. These flagella systems have been mentioned to be associated with bacterial pathogenesis by increasing host cell adhesion ability and swarming motility of *A. hydrophila* [32]–[34]. Moreover, some other structures of *A. hydrophila* such as siderophores, porins, and capsules are also believed to be associated with bacterial pathogenesis. The genes encoding siderophores, high-affinity iron chelating molecules, are present in the *A. hydrophila* ATCC 7966 genome. These molecules have also been reported to affect *Aeromonas* spp. pathogenesis and growth [12], [16], [18]. A pore-forming protein, porin, on the outer membrane of *A. hydrophila* has also been shown to correlate with bacterial resistance to the host immune system. Lack of this protein in O-antigen depleted *A. hydrophila* strains (serum sensitive) increases bacterial serum resistant ability and this results in induction of bacterial survival in human serum [17]. Although the flagella, siderophores, porin, and bacterial capsules have been previously reported to have correlations with *A. hydrophila* pathogenesis, the mutation of these factors in this study did not show any major effects on the virulence of *A. hydrophila* AH-3 in *P. leniusculus* or in *T. molitor* larvae.

This study also used *A. hydrophila* AH-3 strains with mutations in several LPS genes and found that the bacterium completely lost its virulence when the *waa*E gene was mutated. The Δ*waa*E mutant did not cause death in *P. leniusculus* at all and only two *T. molitor* larva died after injection with this mutant strain. The death of *T. molitor* larva, however, could be an effect of injection since some animals also died after being injected with buffer control. The results are correlated with a study performed in mice which showed the LD_50_ for intraperitoneal injection of AH-3Δ*waa*L mutant was approximately 20-fold higher than that of the AH-3 wild type, while the AH-3Δ*waa*E mutant was completely avirulent (data not shown). The results, therefore, indicate that the LPS structure is important for *A. hydrophila* AH-3 virulence especially in the invertebrate models used in this study, and the most important part of this structure is the O:34 antigen LPS and the external core (Figure 1 shows the relevant LPS chemical structures).

Some secretion systems are involved in the release of bacterial toxins and effector proteins, some of which have been reported to play a role in bacterial virulence [6], [13]–[15], [35], [36]. One of the secretion systems which have been widely studied is a type III secretion system (T3SS). This secretion system is present in both clinical and environmental strains of *A. hydrophila* [35]. Mutation of some genes involved in the function of T3SS reduced the virulence of *A. hydrophila* SSU and *A. veronii* in mice and that of *A. hydrophila* AH-1 in blue gourami fishes [13], [36], [37]. Another secretion system which has been recently found in *A. hydrophila* is a type VI secretion system (T6SS) [6], [12]. The study done by Suarez *et al*. [6] showed that mutation of two genes, *vasH* and *vasK*, present in the T6SS gene cluster of *A. hydrophila* SSU resulted in reduction of virulence of this bacterium both *in vitro* and in a mouse model. However, our study shows that T3SS and T6SS are probably not the determinant virulence factors of *A. hydrophila* AH-3 in the experimental models used in this study because mutation of these two secretion systems did not alter the virulence of *A. hydrophila* AH-3 infection in *P. leniusculus* or in *T. molitor* larvae as the AH-3 Δ*waa*E mutant did.

Several toxins like aerolysins, phospholipases, β-hemolysin, HlyA-like hemolysin have been reported to be important for virulence of *A. hydrophila.* Phospholipase and hemolysin genes are present in *A. hydrophila* and their products were reported to have the toxic effects to rainbow trout, crayfish and mice [5], [12], [38]. The *A. hydrophila* AH-3 used in this study has at least the hemolysin genes. *β-hemolysin* and *HlyA-like hemolysin* genes present in both the WT and the Δ*waa*E mutant. The hemolytic, cytotoxic, and caseinolytic extracellular activities showed similar values for the AH-3 Δ*waa*E mutant and the wild type strain (data not shown). Although the WT is virulent whereas the Δ*waa*E mutant is non-virulent in *in vivo* systems, the extracellular products obtained from these two strains showed cytotoxic effects to crayfish hematopoietic cells even though the extracellular products obtained from the *waa*E negative strain showed a slightly lower effect. This indicates that the toxins produced by *A. hydrophila* do not seem to play a major role in *A. hydrophila* virulence *in vivo* in our models. Considering the expression of other virulence factors which might be affected by *waa*E mutation, the AH-3 Δ*waa*E was able to express either polar or lateral flagellum like the AH-3 WT (data not shown). The WaaL LPS mutation in *A. hydrophila* AH-3 downregulated the expression of T3SS [29]. A similar effect was observed for the AH-3Δ*waa*E (data not shown). This indicated that LPS in some cases has a role in regulation of T3SS system [29]. However, T3SS mutation alone has no drastic effects on virulence of *A. hydrophila* AH-3 in our study.

The study on bacterial clearance in *T. molitor* larvae showed different capacity of the worms in handling infection with virulent or non-virulent bacterial strains. The WT continuously grew inside the worms with a statistically significant increase of CFU at 12 h after injection (*P*<0.05) and finally caused death of the worms whereas the non-virulent Δ*waa*E mutant was completely eliminated by *T. molitor* larvae within 12 h. The difference between these two bacterial strains is mainly in their LPS structure. The WT has complete LPS, but the Δ*waa*E mutant which is non-pathogenic lacks the O-antigen and the external core (Figure 1). This indicates that both components of the LPS molecule are important for *A. hydrophila* to escape from the host defense system. This assumption is supported by some previous studies which showed that serum-resistant strains of *A. hydrophila* with complete LPS had high percent survival in non-immune human serum and low binding capacity to complement components [17], [19].

In addition to having different immune-resistant capacity, WT and Δ*waa*E mutant induced different host immune responses. Expression of antimicrobial peptide (AMP) transcripts was investigated in crayfish after injection with WT and Δ*waa*E mutant, and only minor changes in AMP expression was detected. Expression of *Pl*crustin3 and ALF (anti-lipopolysacharide factor) decreased after WT infection but no change in other AMP expressions could be observed. Upregulation of *Pl*crustin1 was observed after Δ*waa*E mutant infection. Expression of this gene have been reported to be upregulated after infection with either pathogenic or non-pathogenic bacteria [39] but an interesting observation in this study is that the upregulation of this gene was observed only following an infection with the non-pathogenic Δ*waa*E mutant.

The AH-3 Δ*waa*E had an ability to induce PO activity in crayfish at a low level but could not activate melanization in *T. molitor* larvae or it might activate melanization to a very low extent which could not be observed. In contrast, the AH-3 WT significantly induced PO activity (*P*<0.05) and subsequent melanization *in vivo*. The AH-3 WT has a complete LPS with O-antigen to protect itself from host defense system [17], [19], [21] whereas Δ*waa*E mutant lacks its defense structure (LPS devoid of O-antigen and external core) and it was cleared very fast from *T. molitor* larvae. Since there were small changes in AMP gene expression but strong activation of the proPO activating system, PO may play a major role as defense mechanism of the host to fight the invading pathogenic *A. hydrophila* AH-3. This hypothesis could be supported by the study in 2007 which showed that PO was important for *P. leniusculus* to defend against *A. hydrophila* B1 infection [25].

In addition to induction of PO activity, the expression level of mRNA of the zymogen proPO and pacifastin was investigated. The expression of proPO was slightly down-regulated in WT infected crayfish, although these animals had a high PO activity, and as expected there was no obvious change in expression of this gene or the PO activity in Δ*waa*E mutant infected crayfish. This higher activity of PO seems to be a result of that the pacifastin transcripts were decreased in the WT infected animals. Pacifastin is an efficient inhibitor of ppA (prophenoloxidase-activating enzyme) and this inhibitor prevents activation of the proPO system. Then if there is no pacifastin, activation of proPO can occur. In a study by Liu *et al.*, [25], knockdown of pacifastin resulted in high PO activity and low bacterial number in crayfish hemolymph at 3 h after bacterial infection. Low expression of pacifastin in WT infected crayfish and high induction of PO activity in this study correlates well with this previous study. The reason for the slight down-regulation of proPO might occur because the proPO system can be activated continuously in the absence of pacifastin and the production of toxic products by PO may have reached levels in which those products are toxic to the animals and as a consequence the proPO transcripts were down-regulated.

Pacifastin of crayfish consist of two subunits containing a proteinase inhibitor light chain and a transferrin heavy chain. These two subunits are encoded by different mRNA transcripts [31], and as described above the expression level of the light chain transcript was decreased in WT infected crayfish but not in Δ*waa*E mutant infected crayfish. In contrast, the heavy chain subunit was down regulated in crayfish infected with either WT or Δ*waa*E mutant. The crayfish pacifastin heavy chain consists of three transferrin lobes of which two can bind iron [31]. Iron is essential for bacterial metabolism and growth and *A. hydrophila* has iron chelating molecules called siderophores for iron acquisition from host iron-binding molecules such as transferrin or lactoferrin [18]. A recent study in fish showed a reduction in serum iron and saturated transferrin following a bacterial infection [40]. Therefore, the down regulation of transferrin subunits of pacifastin might be explained by the low iron level caused by WT or Δ*waa*E mutant AH-3 infection.

In conclusion, mutation in several known virulent factors in an *A. hydrophila* strain in this study provides us a clear picture that O-antigen and external core of the LPS molecule are the *A. hydrophila* AH-3 most important factor for virulence in our animal models. *A. hydrophila* AH-3 WT, which contains a complete LPS core, strongly stimulated the proPO-activating system and melanization in *P. leniusculus* and *T. molitor* larvae, respectively. LPS, therefore, triggers host immune responses and protects bacteria against host defense process which then results in continuous growth of bacteria in the host circulating system and finally leads to death of the host.

## Materials and Methods

### 1. Animals

*Pacifastacus leniusculus* (crayfish) was maintained in aquarium at 10°C with aerated running fresh water. Intermolt crayfish with weights ranging from 30–40 g were selected for the experiments. *Tenebrio molitor* larvae (mealworm) were maintained at room temperature (20–22°C) in beakers placed on the laboratory bench and they were fed with wheat bran. Mealworms with sizes from 2.5–3.5 cm were used for the experiments.

### 2. *A. hydrophila* AH-3 mutant construction

An inner DNA fragment of *vasH, msbB, CirA,* and *uge* was independently obtained from AH-3 chromosomal DNA using appropriate primers and subcloned in the *pir* replication dependent plasmid pSF100 [41] through initial cloning in pGEM-T plasmid. These plasmid constructs (pSF-VasH, pSF-MsbB, pSF-CirA, pSF-Uge, respectively) were used to obtain *vasH*, *msbB*, *cirA*, *uge* deficient mutants from *A. hydrophila* strain AH-3 by a single recombination event leading to the generation of two incomplete copies of wild type genes in the chromosome of these mutants, as previously described [41]. Plasmids pSF-VasH, pSF-MsbB, pSF-CirA, and pSF-Uge, were independently isolated, transformed into *E. coli* SM10 (λ*pir*) [41], and transferred by conjugation from *E. coli* SM10 to the AH-3 rifampin-resistant (Rif^r^) mutant as previously described [17]. Km^r^ Rif^r^ transconjugants arising from the different conjugations using pSF-VasH, pSF-MsbB, pSF-CirA, and pSF-Uge, should contain the mobilized plasmid integrated onto the chromosome by homologous recombination between the wild type gene screened and the plasmid, leading to two incomplete copies of the wild type gene studied (defined insertion mutant). Chromosomal DNA from 10 transconjugants obtained were independently analyzed by Southern blot hybridization with appropriate *vasH, msbB, CirA,* and *uge* DNA probes to obtain the following defined insertion mutants: AH3ΔvasH (T6SS negative), AH3ΔmsbB (lipid alteration in LPS), AH3:: CirA (siderophore negative), and AH3::uge (lacking capsule), as previously described [17].

### 3. Virulence study

This study was performed in both crayfish and mealworm. All mutant and wild type strains of *A. hydrophila* AH-3 were grown at 28°C in tryptic soy broth (Fluka, 20 µg/ml of kanamycin was required for growing all mutant strains except for the T3SS negative one) until they reached a bacteria suspension with an optical density of 0.5–0.6 at 600 nm. The bacterial pellets were then collected and washed 3 times with 0.85% NaCl, and resuspended and diluted in buffer CFS (0.2 M NaCl, 5.4 mM KCl, 10 mM CaCl_2_.2H_2_O, 2.6 mM MgCl_2_.6H_2_O, 2 mM NaHCO_3_, pH 6.8) or in insect Ringer solution (128 mM NaCl, 18 mM CaCl_2_, 1.3 mM KCl, 2.3 mM NaHCO_3,_ pH 7) for injection in crayfish or mealworm, respectively. Bacterial cell concentration for injection was determined and adjusted by performing viable plate counts and is reported as mean of colony-forming unit (CFU) ± SE.

The bacterial suspension (100 µl) was injected into the base of a crayfish walking leg, and 10 µl of the bacterial suspension or insect Ringer solution was injected using Ultra-Fine needle, 31G×8 mm, (BD Micro-Fine) into the abdominal part of *Tenebrio* larvae at the position between the third and the second segments. After injection, animals were normally maintained and fed, and were observed for 7 days. The time that animals died after the injection was recorded and reported as mean ± SE. The mortality of the animal is given as number of animals dead/total number of animals tested (No. dead/No. tested).

### 4. Cytotoxicity of extracellular products of *A. hydrophila* AH-3 WT and Δ*waa*E

Two bacterial strains, *A. hydrophila* AH-3 WT (WT) and *A. hydrophila* AH-3 Δ*waa*E mutant (Δ*waa*E mutant) were chosen for further study because the WT is highly virulent whereas the Δ*waa*E mutant completely lost its virulence. Extracellular products of the bacteria were prepared fresh as described by Jiravanichpaisal *et al*. [5]. Briefly, bacteria, WT and Δ*waa*E mutant, were cultured overnight in 10 ml TSB, and then the culture medium was centrifuged at 8000 g for 10 min at 4°C. The supernatant was collected and sterile filtered through 0.22-µm-pore membranes (Millipore). Then, 10 µl of sterile extracellular products of bacteria or 10 µl of TSB was incubated at room temperature (20–22°C) with freshly isolated crayfish hematopoietic (hpt) cells, which had been prepared as previously described by Söderhäll *et al*. [42]. Briefly, the hpt was dissected out from crayfish and digested with 0.1% of collagenase type I and type IV at room temperature for 45 min, to get single cells. The cells were then washed 3 times with CPBS (10 mM Na_2_HPO_4_, 10 mM KH_2_PO_4_, 0.15 M NaCl, 10 µM CaCl_2_, 10 µM MnCl_2_, 2.7 µM KCl, pH 6.8). The cells were finally resuspended in modified L-15 culture medium [43] and seeded into 96-well plate with the amount of 3×10^4^ cells/well. During the experiments, the morphology of hpt cells was observed every 15 min for 2 h. This experiment was repeated 3 times.

### 5. Bacterial clearance

This experiment was performed in mealworm. *A. hydrophila*, WT (0.4–0.6×10^4^ CFU/animal) or Δ*waa*E mutant (1.8–2.9×10^4^ CFU/animal) were injected into mealworm (n = 3–7) as described above. At 1 h and 12 h after injection, the mealworms were bled, and 10 µl of hemolymph was collected from each individual worm to perform viable plate counts to determine CFU of bacteria in each mealworm. This experiment was repeated 2 times.

### 6. Expression of crayfish antimicrobial peptides (AMPs) and some other immune-related genes after bacterial challenge

Crayfish (n = 3 for each experimental group) was injected with bacteria WT (3.3×10^6^ CFU/animal) or with Δ*waa*E mutant (10.2×10^6^ CFU/animal), and 6 h after injection hepatopancreas and hemolymph were collected and were kept separately. Total RNA was isolated from hemocytes and hepatopancreas using Trizol reagent (Gibco BRL) following the manufacturer's instruction. Each RNA sample was then treated with 2 units of RNase-free DNase I (Ambion) at 37°C for 30 min. Then, RNA was purified again using phenol-chloroform which was followed by ethanol precipitation.

First strand cDNA synthesis was performed using Oligo (dT)_20_ primer and all other reagents were from ThermoScript-PCR kit (Invitrogen) according to manufacturer's instruction. One microgram of RNA obtained from hepatopancreas or 100 ng obtained from hemocytes were used as starting material. cDNAs were then subjected to PCR using the primers shown in Table 3. The 40S ribosomal protein gene was used as an internal loading control for RT-PCR analysis. PCR was performed using the following condition: 95°C for 2 min, 22–38 cycles (see figure 4 and 7 legends for cycles of each gene) of 95°C for 20 s, 58°C for 20 s, 72°C for 30 s, and followed by a cycle of 72°C final extension for 3 min. The PCR products were then subjected to 1.5% agarose gel electrophoresis, followed by ethidium bromide staining and visualized under ultraviolet light.

**Table 3:** Primers used for RT-PCR.

Gene	Primer (5′-3′)	Product size (bp)	Accession No.	References
*Pl*crustin1	GGTAACCATGGCTCGATCAC (F)TGTAATGGTGAGACCGCTCC (R)	368	EF523612	[39]
*Pl*crustin2	CTGCAAGAAGCCTGAAGGTC (F)GCATAACAAGCAAGTCAGCCA (R)	358	EF523613	[39]
*Pl*crustin3	AGCGCCCAGAACACTAACAC (F)GGCAGGTTTGCAGACGTAGT (R)	417	EF523614	[39]
Astacidin2	CCTACAACACCACCATGCGTC (F)CTTGCCAGGTCGGTAGATTGG (R)	140	DQ822206	[39]
ALF	TCCGGAATCTCCTGACAACC (F)TGCGAAGATCTCGGAACTAGGA (R)	451	EF523760	[44]
LGBP	TCATGAGCGCCAAGTTCACC (F)AAGTAGCCATTTGTGCCGCC (R)	529	AJ250128	[45]
40S	CCAGGACCCCCAAACTTCTTAG (F)GAAAACTGCCACAGCCGTTG (R)	360	CF542417	Unpublished data
proPO	TGGCACTGGCATCTCGTTTAC (F)TCCCTCGCTTTCCTGTTCTGAC (R)	406	X83494	[46]
Pacifastin light chain	TGCACCAAGAGGCTTTGTCG (F)TTGGAGCCATCAGTACACACAGC (R)	536	U81825	[31]
Pacifastin heavy chain	TGCAGGGTCGCAAATCTTGCCA (F)ACACTTGCCGCACCTGACTCAA (R)	540	U81824	[31]

### 7. Induction of melanization in mealworms, *T. molitor*

To study the effect of a bacterial infection on melanization, the experiments were performed in mealworm because melanin formation could be seen easily in this animal. *A. hydrophila* WT (1×10^4^ CFU/animal) or Δ*waa*E mutant (1.7×10^4^ CFU/animal) were injected into mealworms (n = 10 each), and melanin formation was observed for 7 days or until the animals died.

### 8. Induction of prophenoloxidase activating system

A study of the effect on the prophenoloxidase (proPO) activating system after bacterial challenge was performed in crayfish because it was more convenient to get enough hemolymph from crayfish for the study when compared to that from mealworm. Activation of the proPO system in crayfish after bacterial challenge was done by preparing hemocyte lysate (HLS) and then determined LPS-activated PO activity in the HLS samples. *A. hydrophila* WT (5.8×10^6^ CFU/animal) or Δ*waa*E mutant (10.1×10^6^ CFU/animal) or CFS was injected into 5 crayfishes each. Six hours after injection, crayfishes were bled and 10 drops of hemolymph from each individual were collected and pooled together in one experimental group and this sample was then centrifuged at 800×g for 20 min at 4°C and the resulting hemocyte pellet was homogenized and then centrifuged again at 16000×g for 20 min at 4°C. The obtained supernatant was HLS and was used for PO activity assay. The assay was performed by incubating 50 µl of HLS, 50 µl of 1 mg/ml LPS (*E. coli* 005:B5; Sigma), and 50 µl of 3 mg/ml L-3,4-dihydroxyphenylalanine (L-DOPA) (Sigma) at room temperature (20–22°C) for 10 min. As a control reaction, sterile distilled water was used instead of LPS. PO activity was determined by measuring the absorbance of dopachrome at 490 nm. The protein content in HLS was determined and PO activity was reported as ΔA490/mg protein/10 min. This experiment was repeated 2 times with a total amount of 10 crayfishes for each experimental group.

### 9. Statistical analysis

All experiments were repeated 2–3 times with 3–10 animals (for each experimental group) used as indicated in each experiment. All animals used in this study were independent from each other, and each animal was used for the measurement or for our study only one time. The T-test was performed when two experimental groups were compared, and One-way ANOVA followed by Turkey's multiple comparison was used for multiple comparisons. The *P*-value <0.05 was considered as a statistically significant difference.
